# Level of maternal antibodies against respiratory syncytial virus (RSV) nucleoprotein at birth and risk of RSV very severe lower respiratory tract infection

**DOI:** 10.1111/irv.13025

**Published:** 2022-10-17

**Authors:** Matthieu Receveur, Michèle Ottmann, Jean‐Marc Reynes, Jean‐François Eleouet, Marie Galloux, Aurore Receveur, Dominique Ploin, Sylvie Fiorini, Nathalie Rivat, Martine Valette, Bruno Lina, Jean‐Sebastien Casalegno

**Affiliations:** ^1^ Hospices Civils de Lyon, Hôpital Femme Mère Enfant Service de Réanimation Pédiatrique et d'Accueil des Urgences Bron France; ^2^ Hospices Civils de Lyon, Hôpital de la Croix‐Rousse Institut des Agents Infectieux Lyon France; ^3^ Université de Lyon, Université Claude Bernard Lyon 1, Faculté de médecine Lyon Est Lyon France; ^4^ Université de Lyon, Université Claude Bernard Lyon 1, Laboratoire de Virologie et Pathologies Humaines Virpath, École Normale Supérieure de Lyon, Centre International de Recherche en Infectiologie (CIRI), Inserm U1111, CNRS, UMR5308 Lyon France; ^5^ Unité Environnement et Risques Infectieux Institut Pasteur, Université Paris Cité Paris France; ^6^ Université Paris‐Saclay, INRAE, Unité de Virologie et Immunologie Moléculaires Jouy‐en‐Josas France; ^7^ OFP/FEMA Pacific Community, SPC Noumea New Caledonia; ^8^ ENTROPIE Université de la Réunion, IRD, CNRS, UMR9220, Université de La Nouvelle‐Calédonie, Ifremer Noumea New Caledonia; ^9^ Hospices Civils de Lyon, Hôpital de la Croix‐Rousse, Institut des Agents Infectieux (IAI), Laboratoire de Virologie, Centre National de Référence des virus des infections respiratoires Lyon France

**Keywords:** infants, lower respiratory tract infection (LRTI), maternal antibodies, nucleoprotein, respiratory syncytial virus, vaccine

## Abstract

**Background:**

The nucleoprotein (N protein) of respiratory syncytial virus (RSV) is a candidate antigen for new RSV vaccine development. The aim of the present study was to investigate the association between maternal antibody titers against the RSV N protein at birth and the newborns' risk of developing very severe lower respiratory tract infection (VS‐LRTI).

**Methods:**

In this single‐center prospective cohort study, 578 infants born during the RSV epidemic season in France were included. Among these, 36 were hospitalized for RSV VS‐LRTI. A generalized linear model was used to test the occurrence of a VS‐LRTI in function of sex, mode of delivery, parity of the mother, type of pregnancy, date of birth in relation to the peak of the epidemic, and antibody titer against N protein.

**Results:**

All cord blood samples had detectable antibodies against N protein. The mean titers were significantly lower in newborns with risk factors for RSV severe LRTI (preterm infants, birth before the peak epidemic, multiparous mother). There was no association between antibody titer against the N protein and a protection against VS‐LRTI.

**Conclusions:**

Further studies are needed to support the hypothesis that transfer of maternal antibodies against the RSV N protein can provide a significant immune protection early in infancy and that N protein candidate vaccine may be a suitable target for maternal vaccine.

## 1 INTRODUCTION

Respiratory syncytial virus (RSV) is the leading cause of bronchiolitis in infants and of pneumonia in children younger than 5 years of age in infants,^1^, ^2^ and in this population, it is responsible each year for approximately 33 million cases of lower respiratory tract infections (LRTI) worldwide.^1^ It is recognized to cause substantial mortality in low‐ and middle‐income countries,^2^ and major lower respiratory tract infection burden in high‐income countries^3^, ^4^; the highest rate of hospitalization is in infants aged less than 3 months.^5^ Despite this, preventive measures are to date limited to the monthly injection of RSV‐specific neutralizing antibodies for children at a high risk of severe complications, the cost of which may be prohibitive.^6^ There are, however, vaccines currently in development that have the potential to be a more affordable option to reduce the worldwide RSV burden.^6^ Most candidate vaccines target the fusion protein, which is an envelope protein highly conserved between different subtypes or RSV, stabilized in its prefusion form.^7^, ^8^ However, RSV nucleoprotein (N protein), which is implicated in nucleocapsid–RNA complex formation that allows RSV replication and transcription,^9^ is one of the most conserved viral proteins among strains^10^ and is also considered as a potential target for candidate vaccines inducing T CD8+ response.^11^, ^12^ These vaccines may be good candidates for maternal immunization strategy, an approach that appears as safe because of the particular vulnerability of infants and their inability to produce effective antibodies.^13^ Such a strategy has already demonstrated its ability to provide a passive humoral protection for infants against tetanus, influenza, and pertussis.^14^ Humoral protection is conferred at birth by the transfer of maternal IgG, the titer of which depends on the maternal level of RSV antibodies and the effectiveness of transplacental RSV antibody transfer from mother to infant.^15^ Although the protection conferred to infants by neutralizing antibodies induced by natural exposure to RSV of mothers has been suggested, published data are conflicting^16^, ^17^, ^18^, ^19^, ^20^; furthermore, there are no published data concerning the protection provided by maternal antibodies against the N protein. The aim of the present study was therefore to investigate the association between maternal antibody titers against the RSV N protein at birth and the newborns' risk of developing RSV VS‐LRTI early in infancy.

## 2 METHODS

### 2.1 Study design

All infants born at the Hôpital Femme Mère Enfant (HFME; part of the university hospitals of Lyon, France) from August 26, 2019, to February 27, 2020 (during the RSV epidemic season in France^4^), with a cord blood sample available were eligible for inclusion in this single‐center prospective observational cohort study. Stillborn children, those living outside the Auvergne‐Rhône‐Alpes region, or those with insufficient sample were excluded. Five hundred seventy‐eight samples were randomly included for analysis.

The administrative registry of all infants born in the university hospitals of Lyon (Hospices Civils de Lyon, HCL) was used to recover the following variables of interest: sex (male/female), month of birth, gestational age (weeks of amenorrhea, WA), maternal parity (primiparity/multiparity), type of pregnancy (simple/multiple gestation), mode of delivery (vaginal birth/caesarean section), and birth weight. Preterms were classified as either “moderate‐to‐late preterm” [from 32 to 36(+6) WA], “very preterm” [28 to 31(+6) WA], or “extremely preterm” [22 to 27(+6) WA], as defined by the World Health Organization (WHO).^21^ Low birth weight was defined as less than 2500 g.^22^

Infants aged 3 months or younger who visited the emergency department of the HFME hospital (either directly or transfer from another hospital) between August 26, 2019, and May 27, 2020, with laboratory‐confirmed RSV bronchiolitis recognized by the International Statistical Classification of Diseases and Related Health Problems (ICD‐10) codes (J21.0, J12.1, and/or J20.5) were identified in the informatics' database of the hospital. A 3‐month follow‐up seemed to be optimal to detect a protection purposed by maternal antibodies as children under 3 months of life experience the greatest risk of hospitalization and mortality^5^, ^23^ and as previous studies have shown that maternal antibody concentrations against RSV decreased between 2 and 6 months,^1^, ^24^ with a half‐life of approximately 27 days.^18^ According to local protocols, all infants hospitalized with an LRTI diagnosis were tested for RSV on a nasopharyngeal sample. Laboratory‐confirmed RSV infection were those diagnosed by real‐time reverse transcriptase (RT)‐PCR, as previously described.^25^ Clinical records were reviewed to further classify these as VS‐LRTI, as defined by the WHO: cough or difficulty breathing, associated with fast breathing or peripheral capillary oxygen saturation (SpO2) < 90%, or inability to feed or unconscious.^13^ The peak of the epidemic was defined as December 9, 2019, based on both pediatric emergency admissions for bronchiolitis and RSV detection data in Lyon, in accordance with data from previous years.

### 2.2 Laboratory procedures

Cord bloods were collected at birth in heparinized tubes, centrifuged, and stored at −20°C until testing. IgG against RSV N protein were measured in 1:100 diluted plasma using an in‐house enzyme‐linked immunosorbent assay (ELISA) performed according to Roux et al^26^ and using recombinant N protein from RSV A long strain and antihuman polyvalent Ig G/A/M peroxidase antibody produced in goat (Sigma‐Aldrich®, Saint‐Louis, MA, USA). A negative control was obtained from the IgM/IgG RSV ELISA kit manufactured by Virotech Diagnostics (EC107.00). The cutoff of our assay was the mean OD value of the negative control tested in duplicate, plus three standard deviations (SD). A sample was considered positive when its OD was above this cutoff. The values of our samples were all above this cutoff. In order to establish a standard curve, eight twofold dilutions (starting at 1:100) of a positive control (BEI Resources NR‐4020, Manassas, VA, USA) were used. Negative control was obtained from the IgM/IgG RSV ELISA kit (Virotech Diagnostics, Dietzenbach, HE, DE). Titers were calculated using a Michaelis–Menten equation applied to the values of the standard curve and expressed as relative unit (RU)/mL.

### 2.3 Statistical analysis

Mean and SD were calculated to describe clinical continuous variables. Concerning antibody titers against RSV N, both mean (SD) and median (interquartile range [IQR]) were calculated. Comparisons between mean antibody titers were tested using a two‐sided parametric Student *t*‐test or an ANOVA for more than two groups. Normality assumption for the antibody titers was visually inspected before performing the ANOVAs and the Student tests. The birth weight and the gestational age were treated as categorical variables. A generalized linear model (GLM) was used to test the occurrence of a VS‐LRTI (with a binomial distribution and a logit link function) in function of sex, mode of delivery, parity of the mother, type of pregnancy, date of birth in relation to the peak of the epidemic (all previous variables are qualitative), and titer antibodies against RSV N (quantitative variable). Preterm infants as well as patients who contracted a VS‐LRTI but for whom the result for RT‐PCR RSV detection was negative were excluded from the GLM. Statistical analyses were performed using R software version 4.0.3.

### 2.4 Ethics

This study was approved by the review board of university hospitals of Lyon is registered on ClinicalTrials.gov (NCT04144816) and was declared to national data protection commission (19‐198).

## 3 RESULTS

### 3.1 Characteristics of the cohort

**FIGURE 1:**
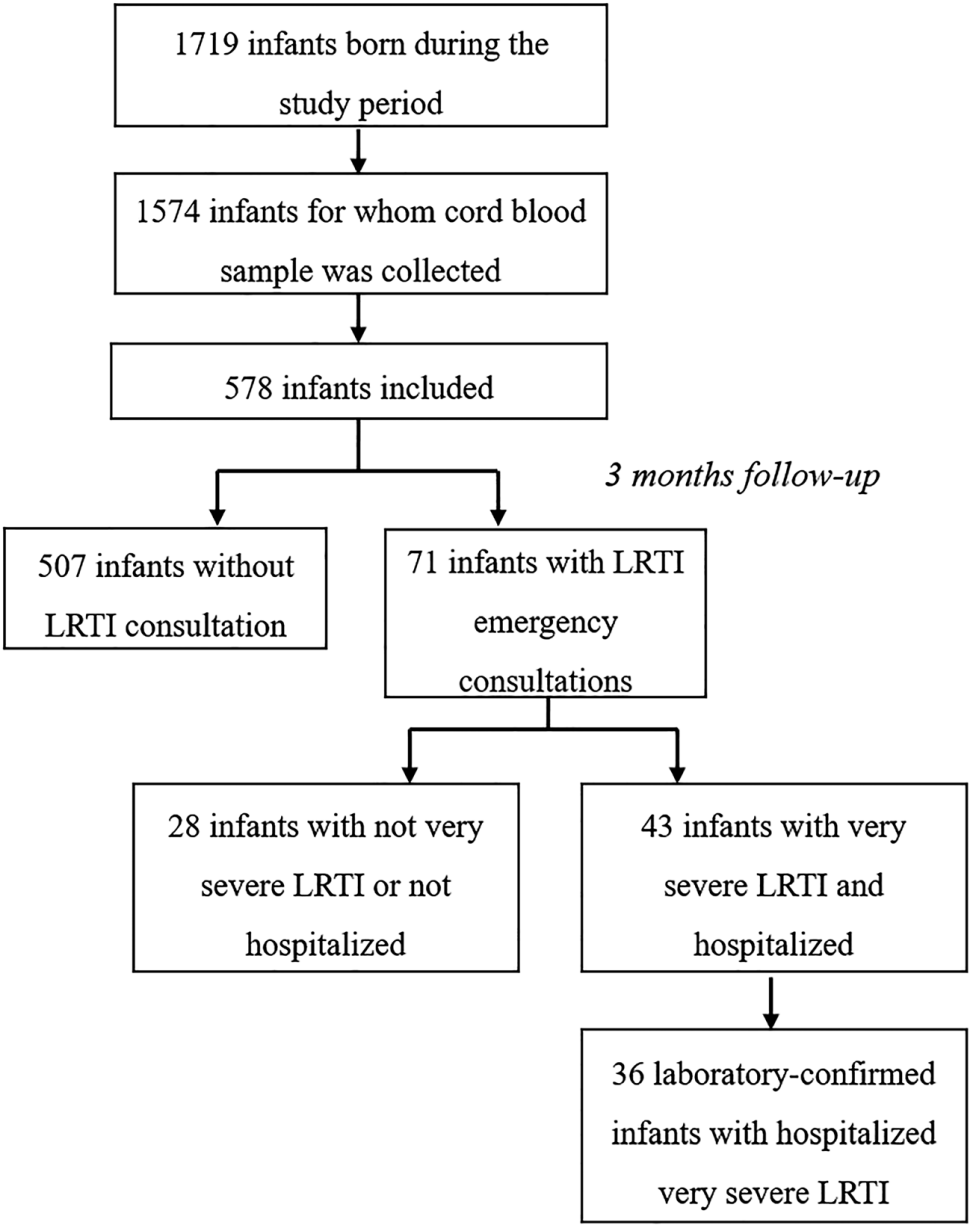
Flowchart. LRTI, lower respiratory tract infection

During the study period, 1719 infants were born at the HFME. Cord bloods were available for 1574 of them, and 578 were randomly included for analysis. Among those included, there were 71 infants aged 0–3 months who visited the emergency department with a diagnosis of LRTI and 43 hospitalized with a diagnosis of VS‐LRTI; of the latter, 36 tested positive for RSV (Figure 1). Two‐thirds of cases (*n* = 24/36) were classified as VS‐LRTI on the respiratory criterion, and a third of cases (*n* = 12/36) were classified as VS‐LRTI only on the “inability to feed” criterion.

The total cohort comprised 578 infants including 53.8% (*n* = 311) boys and 0.9% (*n* = 57) preterm. The mean (SD) birth weight was 3179 (633) g. Delivery characteristic were as follows: 6.1% (*n* = 35) multiple births, 23.0% (*n* = 133) cesarean section, 60.7% (*n* = 351) multiparous mothers, 9.9% (*n* = 57). Among the 36 hospitalized in their first 3 months of life with a laboratory‐confirmed RSV VS‐LRTI infection, 50.0% (*n* = 18) were boys, none were premature, 88.9% (*n* = 32) were born to multiparous mothers, and none were from multiple births (Table 1).

**TABLE 1:** Clinical characteristics of the population

	Total cohort (*N* = 578)	Cases of VS‐LRTI (*N* = 36)
Sex		
Female	267 (46.2%)	18 (50.0%)
Mode of delivery		
Vaginal	445 (77.0%)	29 (80.6%)
Caesarean	133 (23.0%)	7 (19.4%)
Parity		
Monoparity	227 (39.3%)	4 (11.1%)
Multiparity	351 (60.7%)	32 (88.9%)
Gestational age at delivery (weeks of amenorrhea)		
Mean (SD)	38.7 (2.5)	39.1 (1.2)
Term	521 (90.1%)	36 (100.0%)
Moderate‐to‐late preterm	43 (7.4%)	0 (0.0%)
Very preterm	9 (1.6%)	0 (0.0%)
Extremely preterm	5 (0.9%)	0 (0.0%)
Infant birth weight (g)		
Mean (SD)	3179 (633)	3301 (337)
Low birth weight (<2500 g)	65 (11.3%)	0 (0.0%)
Type of pregnancy		
Singleton	543 (93.9%)	36 (100.0%)
Multiple	35 (6.1%)	0 (0.0%)
Date of birth in relation to the peak of the epidemic		
Birth before the peak	277 (47.9%)	31 (86.1%)
Birth after the peak	301 (52.1%)	5 (13.9%)

Abbreviation: SD, standard deviation.

### 3.2 Maternal antibody titers against the RSV N protein

**FIGURE 2:**
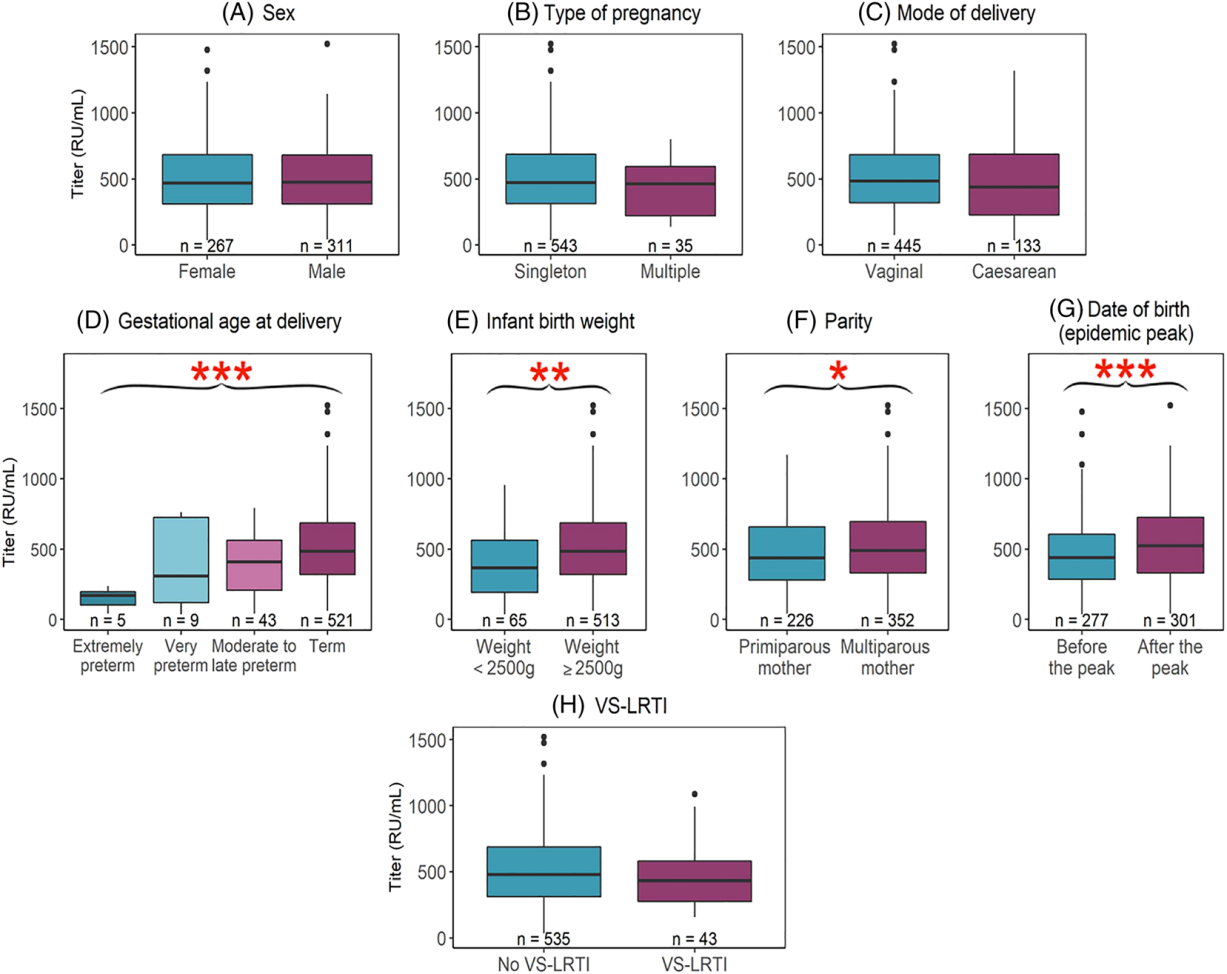
Titers of antibodies against N protein in different subgroups. (A) Sex. (B) Type of pregnancy. (C) Mode of delivery. (D) Gestational age at delivery. (E) Infant birth weight. (F) Parity. (G) Date of birth (epidemic peak). (H) VS‐LTRI. Boxplots present medians and interquartile ranges [IQR], whiskers are 1.5 times the IQR, and outliers are >1.5 times and <3 times the interquartile range beyond end of the box. *P < 0.05, **P < 0.01, ***P < 0.001 in Student's *t*‐test or ANOVA

The mean (SD) value of maternal antibody titers against the RSV N protein among newborns was 502 (255) RU/mL. There was no significant difference in mean values according to sex (Figure 2A), type of pregnancy (Figure 2B), or mode of delivery (Figure 2C). The mean value was significantly different according to the term of pregnancy (*P* < 0.001; Figure 2D); it was significantly lower in preterm newborns (i.e., extremely, very, and moderate‐to‐late combined) than in those born at term (377 vs. 516 RU/mL, *P* < 0.001), in newborns with a low birth weight than in those with a birth weight ≥2500 g (401 vs. 515 RU/mL, *P* < 0.001; Figure 2E), in newborns from primiparous mother than those from multiparous mothers (470 vs. 522 RU/mL, *P* < 0.05; Figure 2F), and in newborns whose birth occurred before the peak of the epidemic than in those born after (458 vs. 542 RU/mL, *P* < 0.001; Figure 2G). There was no significant difference in mean antibody titers between infants who experienced a VS‐LTRI (irrespective of RSV detection) and those who did not (466 vs. 505 RU/mL, *P* = 0.37; Figure 2H).

### 3.3 Prediction of VS‐LRTI using clinical variables and serological status

Preterm infants were excluded from the GLM given that a 3‐month follow‐up was not sufficient to analyze their risk of being admitted to hospital for VS‐LRTI (they might have been hospitalized since birth at 3 months of age). In multivariate analysis, maternal multiparity (relative risk, RR: 2.34, 95%CI [1.58; 3.01]) and a date of birth before the peak of the epidemic (RR: 2.84, 95%CI [2.08; 3.59]) were significantly associated with the occurrence of VS‐LRTI; there was no significant association between antibody titer and VS‐LRTI (Table 2).

**TABLE 2:** Multivariate analysis of the factors associated with VS‐LRTI

	Level	Relative risk (RR) of VS‐LRTI	[95% CI]	*P*‐value	Significance
Sex	Female	Ref	[0.34; 1.45]	0.75	‐
	Male	0.89			
Type of pregnancy	Singleton	Ref		0.99	‐
	Multiple	0.14	[−1.42; 1.40]		
Mode of delivery	Vaginal	Ref		0.79	‐
	Caesarean	0.88	[0.14; 1.62]		
Parity	Monoparity	Ref		<0.01	**
	Multiparity	2.34	[1.58; 3.01]		
Date of birth in relation to the peak of the epidemic	Birth after the peak	Ref		<0.001	***
	Birth before the peak	2.84	[2.08; 3.59]		
Titer of antibodies	Slope	−0.00049	[−0.00167; 0.000692]	0.50	‐

Abbreviations: 95% CI, 95% confidence interval; VS‐LRTI, very severe low respiratory tract infection.

^**^ *P* < 0.01.

^***^ *P* < 0.001. Relative risk and p values are calculated using a generalized linear model.

## 4 DISCUSSION

In the present study, no association was found between antibody titer against the RSV N protein measured in cord blood and a protection against RSV VS‐LRTI. This result differs from the largest cohort studies that analyzed cord blood titers; these studies, conducted in Denmark, Mali, and on American Indian infants, found that a higher concentration of neutralizing antibodies at birth was associated with a lower risk of contracting an RSV LRTI and of being hospitalized for this.^14^, ^15^, ^23^ Considering the power of the present study, the sample size is comparable with the study reported by Buchwald et al (587 infants),^18^ and greater than that reported by Eick et al (372 infants),^16^ which both concluded to a protection conferred by neutralizing antibodies (whereas the study conducted in Alaska [155 infants] and Kenya [90 infants] did not find any association).^17^, ^18^ For the present study, owing to the lack of data available regarding antibody titers against RSV N protein, there was no determination of number of subjects required; the data provided herein will therefore help for the power calculation in future studies.

It is of note that the mean antibody titers against RSV N protein at birth were lower in premature infants, those with a low birth weight, born to a primiparous mother, and born before the peak of the epidemic. This profile is not surprising; for instance, it is well known that majority of transplacental transfer of IgG occurs during the third trimester of pregnancy and that preterm newborns have a compromised passive immunity.^27^, ^28^, ^29^ Furthermore, multiparous mothers are likely to have been exposed to RSV through household transmission from a co‐occupant aged between 2 and 13 years,^30^ as well as those delivering at the end of the epidemic season. These factors are also known to be risk factors for severe RSV infection.^31^, ^32^, ^33^ The multivariate analysis indicates that this risk is mostly mediated through another pathway that the lack of RSV N antibodies.

The study does have some limitations. The most important is that only patients admitted to the hospital they were born in were considered for the analysis of VS‐LRTI; thus, any cases visiting elsewhere may have been missed. However, there are few other pediatric emergency departments in the region; the HFME university hospital is the largest pediatric hospital in the area and the only one with an intensive care unit; thus, it is likely that most cases of RSV VS‐LRTI occurring before 3 months of age were captured from either direct admission or transfers from other hospitals. Although VS‐LRTI before 3 months of age is a relevant outcome, the stringency of this definition reduces the probability of observing an outcome in some less frequent groups. As a consequence, we were not able to test the impact of prematurity and low birth weight in the multivariable analysis.

Furthermore, detection used ELISA that allows standardization of result but does not quantify neutralizing activity. However, a neutralization assay would not have specifically detect antibodies against N protein that were investigated herein. Finally, the severity of LRTI episodes might have been overestimated; a third of cases (*n* = 12/36) were classified as VS‐LRTI only on the “inability to feed” criterion, and patients hospitalized for LRTI were very likely to have enteral nutrition, sometimes for less than 24 h (*data not shown*). However, it is unlikely to have led to a differential selection according to the antibody titer level, and the classification of all episodes of LRTI according to the WHO standardized severity criteria allows comparison with future studies. Finally owing to the lack of data on antibody titers against RSV N protein, we were not able to determinate the number of subjects prior to the study. It is not possible to conclude here to the absence of protection confer by the N‐antibody against severe LRTI. Next studies on RSV N antibody level at birth should considered adding others RSV antibody assays and included more patients.

In conclusion, in this study, we did not find any significant association between anti‐N antibody titers at birth and VS‐LRTI occurrence the first 3 month of life. Further studies are needed to support the hypothesis that transfer of maternal antibodies against the RSV N protein can provide a significant immune protection early in infancy and that N protein candidate vaccine may be a suitable target for maternal vaccine.

## AUTHOR CONTRIBUTIONS

**Michèle Ottmann:** Conceptualization; resources. **Jean‐Marc Reynes:** Conceptualization; resources. **Jean‐François Eleouet:** Conceptualization; data curation. **Marie Galloux:** Conceptualization. **Aurore Receveur:** Formal analysis; methodology; software. **Dominique Ploin:** Conceptualization; supervision. **Sylvie Fiorini:** Formal analysis. **Nathalie Rivat:** Formal analysis. **Martine Valette:** Conceptualization. **Bruno Lina:** Conceptualization; validation. **Jean‐Sebastien Casalegno:** Conceptualization; methodology; project administration; supervision.

## CONFLICTS OF INTEREST

None.

### PEER REVIEW

The peer review history for this article is available at https://publons.com/publon/10.1111/irv.13025.

## Data Availability

Data available on request due to privacy/ethical restrictions.
